# Tangrams: a simple visual tool for communicating the complexities of professionalism

**DOI:** 10.12688/mep.17558.2

**Published:** 2022-05-25

**Authors:** Hilary Neve, Sally Hanks

**Affiliations:** 1 Peninsula Medical School, University of Plymouth, Plymouth, UK; 2 Peninsula Dental School, University of Plymouth, Plymouth, UK

**Keywords:** Professionalism, Analogy, Professional Identity, Hidden curriculum, Capability, Complexity

## Abstract

Professionalism is vital for high quality healthcare and fundamental to health profession education. It is however complex, hard to define and can be challenging to teach, learn about and assess.

We describe the development and use of an innovative visual tool, using a tangram analogy, to introduce and explore core professionalism concepts, which are often troublesome for both learners and educators. These include the hidden curriculum, capability, professional identity and the difference between unprofessionalism and high professional standards.  Understanding these concepts can help individuals to see professionalism differently, encourage faculty to design professionalism programmes which focus on professional excellence, support assessors to feel more confident in identifying and addressing underperformance and facilitate learners to appreciate the complexity and uncertainty inherent in professionalism and to become more alert to the hidden curriculum and its potential impact.

We have used the tangram model to educate for professionalism in multiple contexts with learners and educators. Participants regularly report that it leads to a deeper understanding and important new insights around professionalism and helps them identify ways of changing their practice.  We believe this approach has relevance across the health professions and suggest ways it could be further developed to explore wider professionalism issues such as reflective practice, resilience and teamworking.

## Introduction

Professionalism is vital for high quality healthcare and is an established element of most health professional curricula. Yet its ‘extraordinarily complex’ ^1^ (p.25), multi-dimensional nature and the lack of a single, all-encompassing definition ^2^ means that professionalism can feel intangible and difficult to both learn about and teach. 

We are two clinical academics who have taught, assessed and led the development of professional curricula for 19 (HN) and 11 (SH) years. We have also co-chaired national councils for undergraduate teachers of professionalism in medicine and dentistry, respectively. We have found that promoting a single definition of professionalism is usually unhelpful, particularly as different definitions and models of professionalism found in the literature seem to resonate with different people. In our education work, we explain this and provide students with links to a range of professionalism frameworks and definitions to explore for themselves. What we have developed over the years, however, is a visual tool for communicating, in a meaningful way, those professionalism concepts which we believe are crucial for learners and teachers of professionalism to understand but which, in our experience, can be troublesome for them to grasp ^3^. This tool and set of concepts now underpin our professionalism education work. The tool uses analogy which can help facilitate understanding of difficult concepts ^4^. It is based on the tangram, a Chinese puzzle which traditionally consists of seven flat polygons. The individual pieces can be fitted together in different ways to make a diverse range of shapes. We use this tool in a range of ways to explore professionalism concepts, ensuring our discussions link to the literature and to learners’ own professional experiences.

## Tips

### 1. Promoting meaningful understandings of professionalism and how it develops over time

**Figure 1.**
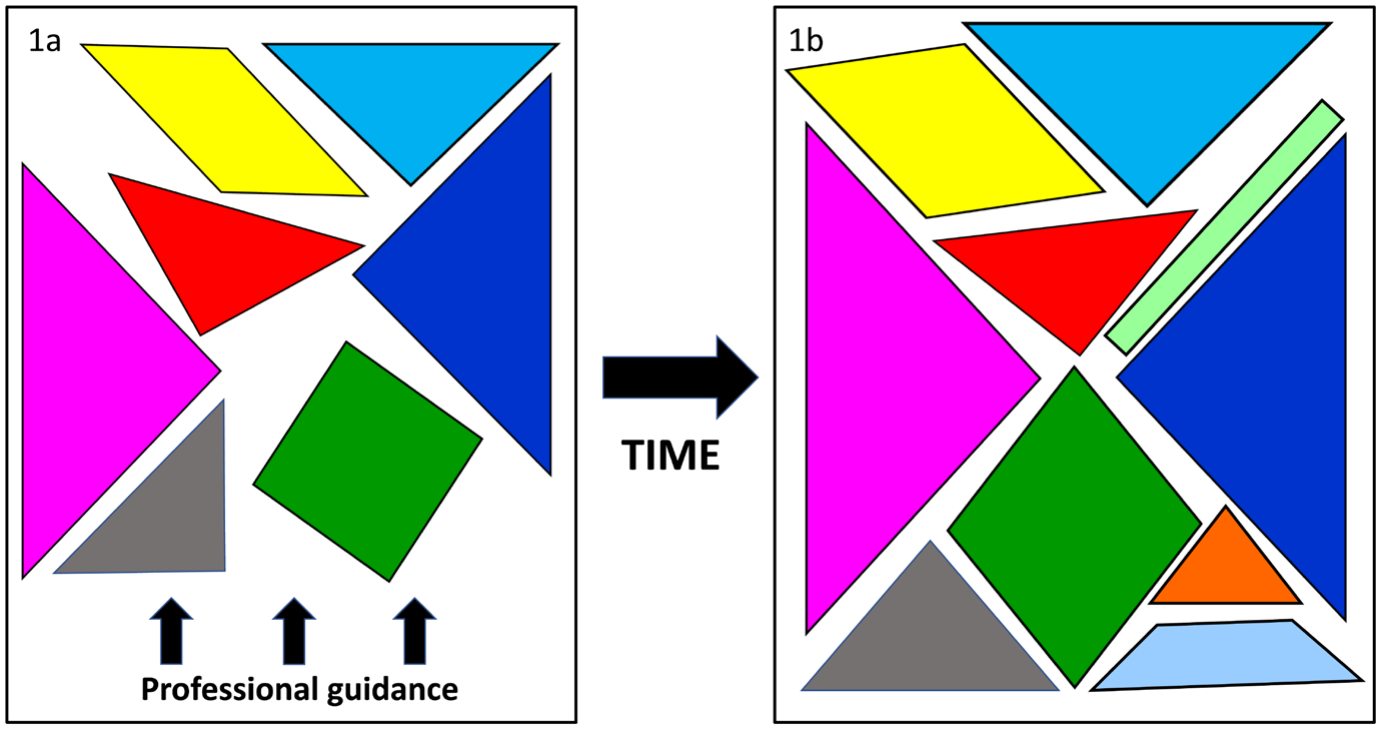
Elements of professionalism as detailed in professionalism guidance ( **1a**). Learners will add to and extend these in response to personal experiences ( **1b**).

We use tangrams to discuss those core elements of professionalism which are often detailed in professional guidance for each health professional group ( Figure 1a). These usually include knowledge (e.g. of ethical principles), skills (e.g. ability to work well as a team) and attitudes/values (e.g. honesty, self-awareness). While these elements are considered core, as learners progress through their courses and professional lives, they will develop and expand their understandings of professionalism. Figure 1b illustrates how learners’ understanding of each of the core elements may deepen and change over time. We breach the traditional 7-piece nature of tangrams to demonstrate how, as students develop and their knowledge and experience of professionalism evolves, they will both refine the existing elements and add new pieces.

### 2. Helping learners understand the concept of ‘professional identity’

A vital element of health profession education is to support the development of each learner’s unique professional identity ^5^. As they learn and interact with others (including patients, role models ^4 ^and teachers) students gradually develop a set of beliefs and values that underpin their individual evolving identity – how they think, feel and view themselves as a health professional. 

**Figure 2.**
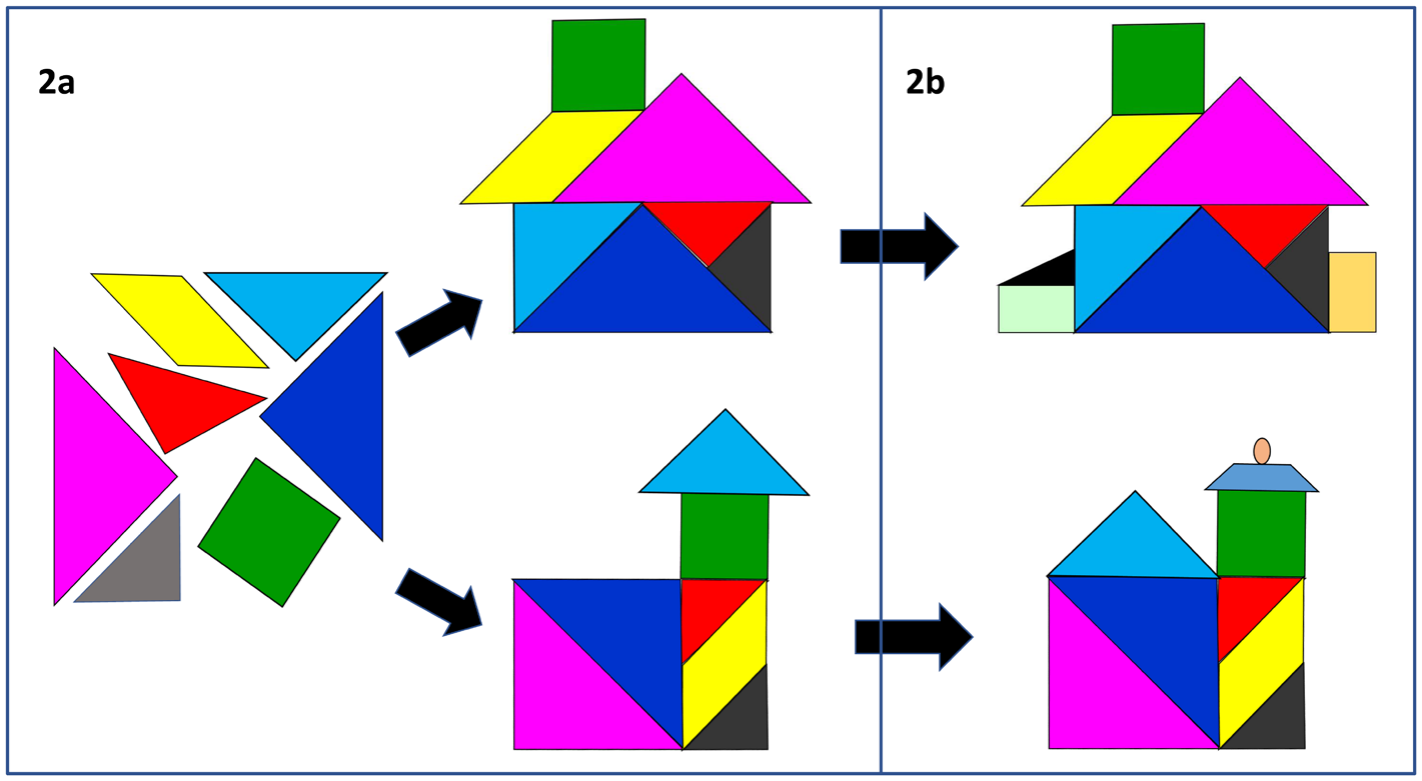
Each individual will develop their own unique professional identity ( **2a**) which will continue to develop over time ( **2b**).

We find learners and teachers often struggle with the notion of ‘professional identity’ and use tangrams to help explain how, as students socialise into communities of healthcare practice ^5^, they will start to appreciate the inter-relatedness of the different aspects of professionalism. They will each make sense of, and fit the core elements together, in a distinctive way that feels right to them ( Figure 2a), influenced, inevitably, by their personal background, values and culture ^6^. The house tangram ( Figure 2a) can be particularly helpful here, illustrating how, through reflection and planning, individuals become the architect, builder and decorator of their own professional identity; renovating, modernising and adding to their house ( Figure 2b) as their career develops. We recommend combining the tangram model with strategies shown to help students to develop their own complex, embodied understandings of professionalism, such as reflecting on personal experiences and professionalism issues in small groups ^1, 7^.

### 3. Communicating a key concept: professionalism is not the opposite of un-professionalism

Our experience is that faculty can spend considerable time identifying learners who are unprofessional, remediating and addressing fitness to practise issues. While this is an important element of professionalism, it can lead to the assumption that any student or health professional about whom there are no concerns, is therefore ‘professional’. Understanding that professionalism involves much more than ***not being unprofessional*** is in our experience, one of the most important, but troublesome, concepts. Failure to grasp this may mean educators focus their efforts on ensuring students meet minimum professionalism standards rather than gaining high-level knowledge and expertise (such as dealing with ambiguity, mitigating personal bias, managing stress and handling challenging conversations). Assessors may ‘fail to fail’ ^8^ a student who is friendly, smart and polite because they are not ‘unprofessional’, even when they do not meet the standards of professionalism required in that setting. Both assessors and students may worry that a negative professionalism grade labels a student as unprofessional. This can lead to student distress and further increase assessors’ reluctance to identify professionalism underperformance.

**Figure 3.**
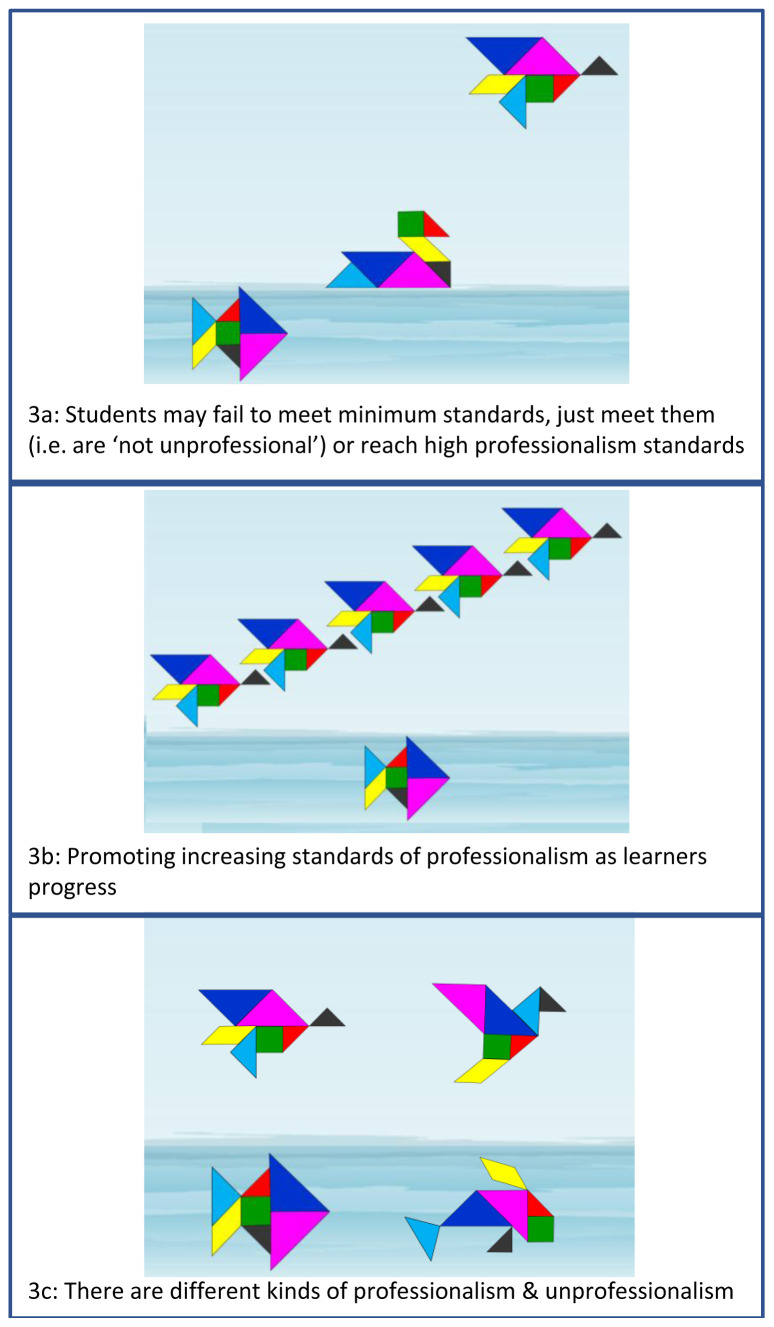
Individuals may meet unsatisfactory, minimum or high standards of professionalism.

It is therefore crucial that students and teachers understand that failing to meet high professionalism standards is not the same as being unprofessionalism and we use tangrams with both groups to explore this. In Figure 3a, the fish represents an individual who is swimming below the expected level and would be considered ‘unprofessional’. The duck on the surface meets minimum standards, so is not unprofessional, but may need support to meet the level expected of them. The flying bird represents an individual demonstrating high standards of professionalism. It is notable that professional bodies, such as the UK General Medical Council, now highlight the importance of students ‘going above and beyond’ ^9^ (p.7) and aiming for professional excellence.

In our medical and dental schools, as students progress through their undergraduate years, they are supported to develop their professionalism, guided by professional criteria which build year on year ( Figure 3b). Students who are unprofessional are always found below the surface, although there can be various kinds of unprofessionalism (and types of fish), just as there are various types of bird who float or fly ( Figure 3c). Given the context-specific nature of professionalism ^2^ it is also possible for an individual to be a bird, or duck, in one setting and a fish in another.

### 4. Raising awareness of the hidden curriculum and how it can influence professionalism

**Figure 4.**
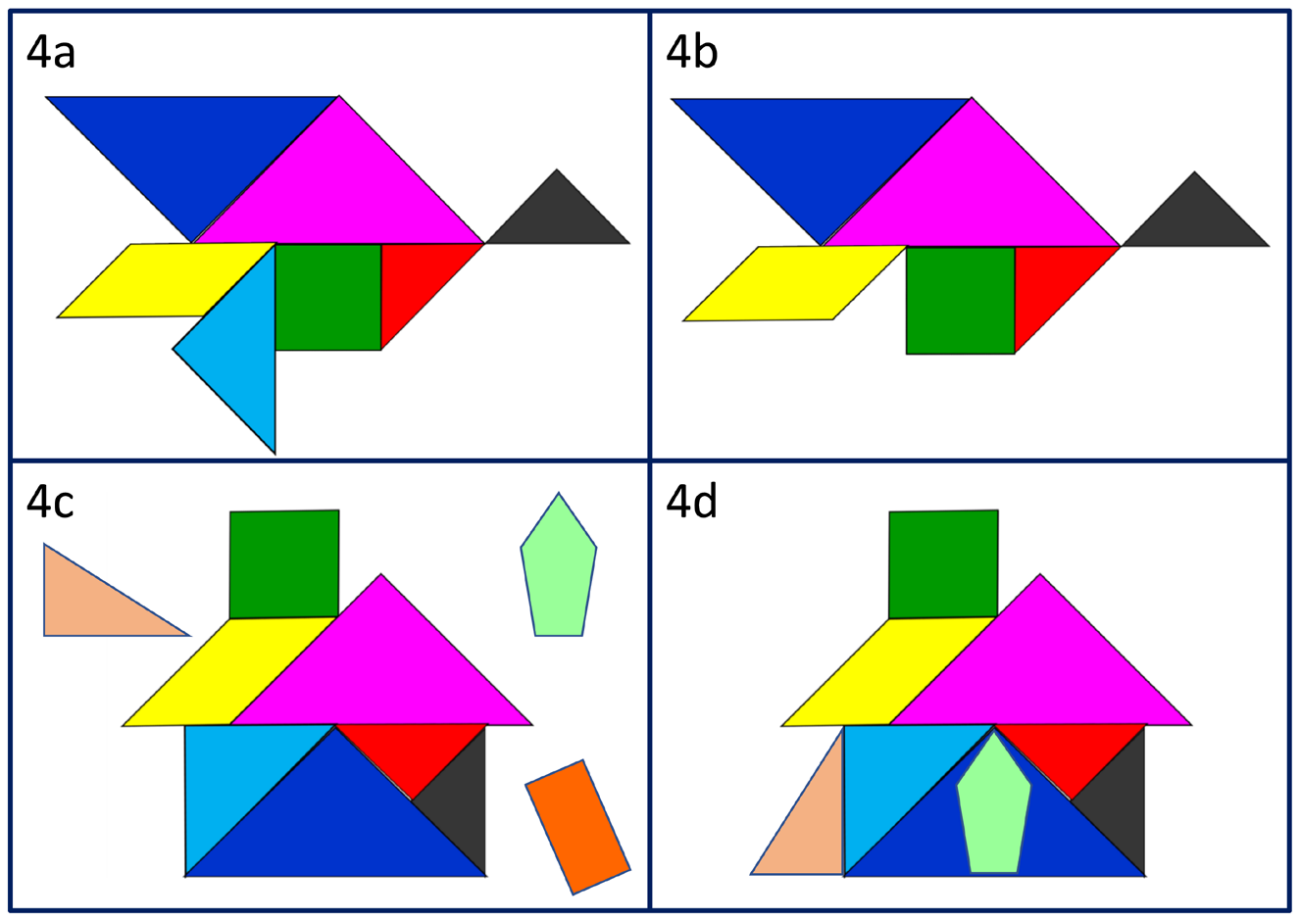
Reflecting on the hidden curriculum: noticing missing elements of professionalism ( **4a** & **4b**); noticing new elements ( **4c**) and choosing which to take on board ( **4d**).

Historically it was often assumed that learners would absorb professionalism by interacting and learning from role models ^10^. We now know that this is not straightforward, that the hidden curriculum can have a powerful influence on the development of learners’ professional identify, across the health professions and across the spectrum of training ^11, 12^. We address this by making the hidden curriculum explicit to learners ^13^. When reflecting on a negative experience, we use the tangram analogy to help learners identify which professionalism element was absent or inadequate, and to consider the impact of this. In Figure 4a and Figure 4b for example, the missing or under-developed wing may mean the bird struggles to fly, or even falls, putting patients, colleagues and the individual themselves at risk. When exploring a positive experience, we support learners to consider what new or additional elements of professionalism they observed ( Figure 4c), how they were useful and whether and how they want to incorporate these into their own professional practice ( Figure 4d).

### 5. Helping learners appreciate the uncertain, complex and changing nature of professionalism

**Figure 5.**
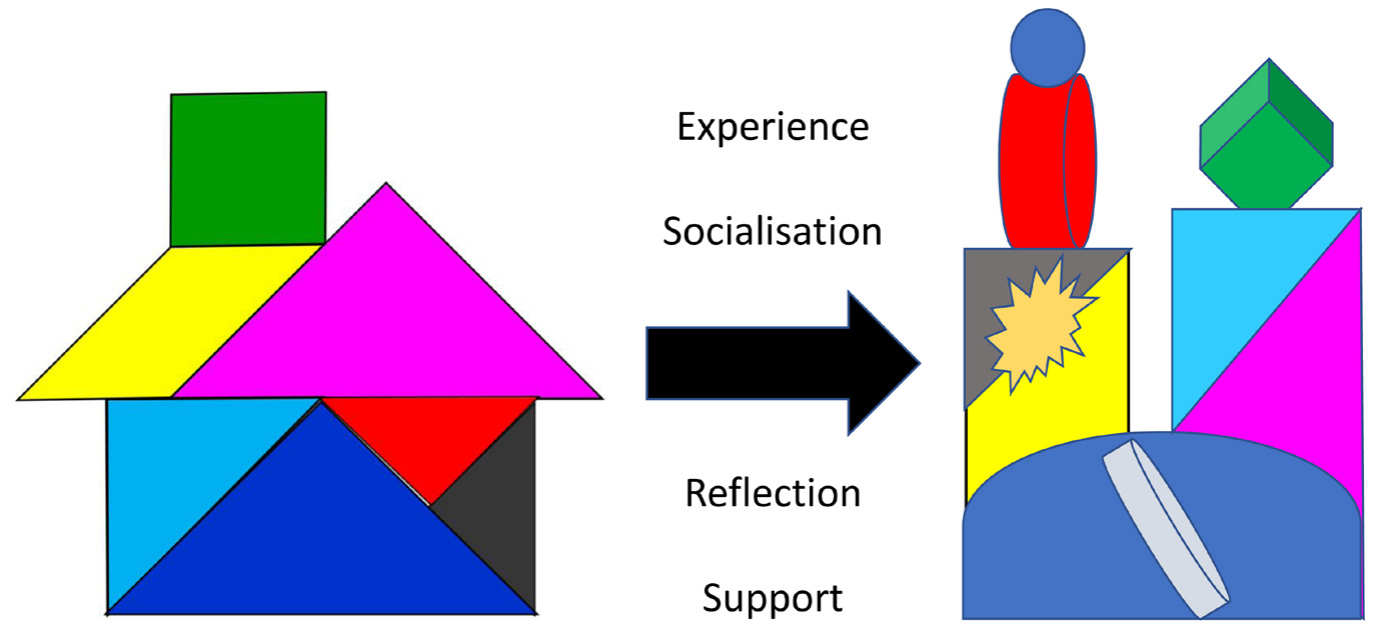
Appreciating the complex, uncertain, multidimensional and changing nature of professionalism.

Working effectively in complex, uncertain and constantly changing healthcare environments requires learners to be self-aware and to appreciate that each situation is different, with no single right answer. We use tangrams to explore capability: the ability to integrate and adapt various combinations of professional skills, knowledge and attributes in each new context ^14^. It is crucial that individuals can ‘shapeshift’ during their careers, redesigning their tangram houses in response to learning and ever-changing contexts ( Figure 5). They may need to expand some elements or develop new areas of professionalism ( Figure 2b) to meet new challenges and work effectively with colleagues who may have different interpretations or cultural understandings of professionalism ^15^. As learners recognise the uncertain, multidimensional, complex nature of professionalism ^16^ ( Figure 5), they can experience significant shifts in how they see themselves as health professionals. This can be troublesome and unsettling and we discuss with educators our vital role in supporting learners through this process.

## Discussion

Since developing the tangram analogy model, we have shared this with students and educators in many settings. The model has not been formally evaluated. However, in their end of workshop evaluations participants frequently describe gaining a greater understanding of the concept of professionalism and its different areas, as well as of the hidden curriculum. Some report that understanding the concept of professional identity means they are less afraid to work and act in a slightly different way to colleagues, or that they will think about professionalism more explicitly when faced with challenging situations. Educators often say they feel better prepared for the challenges of teaching professionalism and how to be a better role model and will be more critical when assessing professionalism.

There are a range of existing models which can help educators when talking with students about definitions of professionalism, and others which can facilitate learners’ understanding of specific professionalism concepts such as reflection or the hidden curriculum. However the agile nature of the tangram model enables it to be used, in a range of ways, to promote understanding of multiple professionalism concepts. In addition, being visual and colourful helps ensure the model is memorable.

There are limitations to the tangram model, in particular how the small number of professionalism pieces and the well-defined shapes that fit neatly together may not reflect the real world. Explicitly exploring these issues in training sessions often leads to further useful debate, for example about the value inherent in terms such as ‘fluffy’ or ‘soft skills’ when describing professionalism. Participants also suggest new ways of using tangrams. For example, using the house tangram to explore how individuals who are stressed or lack motivation may not re-design or update it in response to change, putting patient care and safety at risk – and the importance of critical reflection and developing lifelong learning skills and resilience to prevent this. A shoal of tangram fish or flock of birds can trigger discussion about collusion, the risk of unprofessionalism going unnoticed as well as the benefits of a strong team in supporting each other. We always welcome new ideas!

## Conclusion

The tangram analogy offers an innovative, but practical visual tool for facilitating learning and exploration of fundamental, but often troublesome, professionalism concepts. Learners report that the tool helps their understanding of professionalism and could significantly influence their practice. We believe the analogy is relevant and could be useful to students, clinicians and educators across the health disciplines.

## Data availability

No data are associated with this article.
