# Supplementary material for: Tangrams: a simple visual tool for communicating the complexities of professionalism
Source: MedEdPublish (2016). 2022 May 25;12:2. Originally published 2022 Jan 14. [Version 2] doi: 10.12688/mep.17558.2 (PMC9370087; doi:10.12688/mep.17558.2)
Supplement: Peer review history

# Peer review: Reviewer response for version 2

**DOI:** 10.21956/mep.20526.r32039

**Published:** 2022-05-26

**Authors:** Simon Tso

**Affiliations:** 1 Jephson Dermatology Centre, South Warwickshire NHS Foundation Trust, Warwickshire, UK

In this revised manuscript, the authors have addressed the queries I made previously.

If evidence from practice is presented, are all the underlying source data available to ensure full reproducibility?

Yes

Is the topic of the practical tips discussed accurately in the context of the current literature

Yes

Are all factual statements correct and adequately supported by citations?

Yes

Are the conclusions drawn balanced and justified on the basis of the presented arguments?

Yes

Are arguments sufficiently supported by evidence from the published literature and/or the authors’ practice?

Yes

Reviewer Expertise:

Disability, transition

I confirm that I have read this submission and believe that I have an appropriate level of expertise to confirm that it is of an acceptable scientific standard.

# Peer review: Reviewer response for version 1

**DOI:** 10.21956/mep.18833.r31899

**Published:** 2022-05-06

**Authors:** Faraz Khurshid

**Affiliations:** 1 School of Medicine, Medical Education Unit, Campbelltown Campus, Western Sydney University, Penrith, NSW, Australia

I am extremely grateful to the editorial team for their generous invitation of reviewing this interesting article about ‘Tangrams & Professionalism’ by Hilary Neve and Sally Hanks. Professionalism as always seems to me as a very dry and expanded phenomenon that is not always easy to teach, learn and assess as rightly mentioned by the authors.

 First of all, I acknowledge the authors for their interesting efforts in bringing the elements of vividness and colour to this remarkably complex phenomenon of professionalism. The visual approach not only simplifies the inherent complexity of this universal phenomenon but may also help to improve the communication about the unfathomable aspects of the phenomenon across a range of stakeholders. The consistent line of reasoning is well supported by analogies; however, it is also important to see its practical implications of it across various disciplines in health or allied health sciences.

 Moreover, I was also curious to know how the authors shared and took the feedback of students and educators about this tangram analogy model. A brief insight into the methodology may give a clue to the ‘trustworthiness’ of the data collection process. Having my interest in graphic medicine and concept animation, I am wondering if we can evolve this model into comic strips or animation.

If evidence from practice is presented, are all the underlying source data available to ensure full reproducibility?

Partly

Is the topic of the practical tips discussed accurately in the context of the current literature

Yes

Are all factual statements correct and adequately supported by citations?

Yes

Are the conclusions drawn balanced and justified on the basis of the presented arguments?

Yes

Are arguments sufficiently supported by evidence from the published literature and/or the authors’ practice?

Yes

Reviewer Expertise:

Threshold concepts, Concept Animation, Graphic Medicine, Health Professions Education

I confirm that I have read this submission and believe that I have an appropriate level of expertise to confirm that it is of an acceptable scientific standard.

# Response

**Published:** 2022-05-18

**Authors:** hilary Neve

**Affiliations:** University of Plymouth, UK

Thank you for your positive feedback on our paper and your support for our ‘colourful’ visual approach. In response to your question about methodology, feedback was taken after each of the workshops where we have shared this model. A formal evaluation is not required for a Practical Tips article but we do agree it would add robustness and do hope to undertake such a study in the future. We love the idea of a comic strip or animation, please do contact us if you would like to work with us on this.

# Peer review: Reviewer response for version 1

**DOI:** 10.21956/mep.18833.r31871

**Published:** 2022-04-19

**Authors:** Simon Tso

**Affiliations:** 1 Jephson Dermatology Centre, South Warwickshire NHS Foundation Trust, Warwickshire, UK

Thank you for inviting me to review the manuscript entitled 'Tangrams: a simple visual tool for communicating the complexities of professionalism’. This is a short and succinct manuscript written in the format of a practical tips article describing the authors’ construct and own experience of using tangrams as a visual tool to facilitate communication/education about the complex concepts of professionalism in health professional education settings.

 Professionalism is an important and complex health professional education issue that is often difficult to communicate and teach. Thus, the issues discussed in the manuscript are topical and highly relevant to learners and staff in clinical and academic settings. The authors briefly described their educational credentials, their reflexivity in the definition of professionalism, and their construct of the visual tool, which is very helpful to readers to interpret the contents and relevance of the manuscript to their own professional practice.

 The manuscript further provided (in increasing level of difficulties) various examples of how tangrams can be used to facilitate learners/educators to communicate complex concepts in professionalism. The manuscript has a good flow to it with appropriate literature cited throughout the manuscript. 11 of 16 references used in this manuscript were dated within the past 10 years.

 Overall, this is a useful paper offering readers an alternate way to communicate complex concepts in professionalism and would be a welcomed addition to the academic literature.

 However, I would be grateful if the authors and readers could consider my comments below.

**Major comments:**

- Abstract: ‘ *Participants regularly report that it leads to a deeper understanding…*’, and Discussion, 1 ^st^ paragraph: ‘ *…participants frequently describe gaining a greater understanding of the concept of professionalism… when assessing professionalism.*’
  The authors made the assertions in the abstract and discussion sections that the visual model had been helpful in various ways, however, it remained unclear whether the full spectrum of their evaluation findings (including negative results) had been included in this manuscript to enable readers to interpret the utility of the model and potential for bias.
- The manuscript provided readers with a very helpful visual model to use. However, it is unclear if alternate models exist. It is my opinion that the discussion section could mention or briefly discuss these alternate models (or the lack of) from the literature in order to provide a more balanced discussion on how to teach/communicate complex concepts in professionalism.

**Minor comments:**

- Discussion, 1 ^st^ paragraph, line 1 stated ‘ *and educators in many settings In their end of session*’ - missing full stop. Consider changing to ‘ *and educators in many settings. In their end of session*’.

If evidence from practice is presented, are all the underlying source data available to ensure full reproducibility?

Yes

Is the topic of the practical tips discussed accurately in the context of the current literature

Yes

Are all factual statements correct and adequately supported by citations?

Yes

Are the conclusions drawn balanced and justified on the basis of the presented arguments?

Yes

Are arguments sufficiently supported by evidence from the published literature and/or the authors’ practice?

Yes

Reviewer Expertise:

Disability, transition

I confirm that I have read this submission and believe that I have an appropriate level of expertise to confirm that it is of an acceptable scientific standard, however I have significant reservations, as outlined above.

# Response

**Published:** 2022-05-18

**Authors:** hilary Neve

**Affiliations:** University of Plymouth, UK

Thank you very much for your insightful review. We are pleased that you consider the article topical, highly relevant, and very helpful to readers. In response to your comments:

1. **Lack of a full spectrum of evaluation findings including negative results**.
  This model has not been formally evaluated and the feedback we include comes from post-workshop comments. For this reason, we submitted the paper as a 'Practical Tips' article which does not require formal data but does need to be supported appropriately by the literature, which as you highlight, we have done. In the light of both our reviewers’ comments we will clarify this in the article.
2. **The lack of discussion of alternate models**
  We do briefly mention the existence of other models and definitions of professionalism in the introduction but appreciate that this could be revisited in the discussion. We are not aware of alternative models which are agile enough to facilitate understanding of a variety of specific professionalism concepts. We will make this clearer in the discussion. 

Thank you for highlighting these areas. We will also take these into account when developing our work with the tangram model.
